# Powerful CRISPR-Based Biosensing Techniques and Their Integration With Microfluidic Platforms

**DOI:** 10.3389/fbioe.2022.851712

**Published:** 2022-02-23

**Authors:** Bing Chen, Ya Li, Feng Xu, Xiaonan Yang

**Affiliations:** ^1^ Department of Gastroenterology, The First Affiliated Hospital of Zhengzhou University, Zhengzhou, China; ^2^ Institute of Intelligent Sensing, Zhengzhou University, Zhengzhou, China

**Keywords:** CRISPR/Cas systems, biosensor, microfluidic techniques, isothermal amplification, nucleic acid detection

## Abstract

In the fight against the worldwide pandemic coronavirus disease 2019 (COVID-19), simple, rapid, and sensitive tools for nucleic acid detection are in urgent need. PCR has been a classic method for nucleic acid detection with high sensitivity and specificity. However, this method still has essential limitations due to the dependence on thermal cycling, which requires costly equipment, professional technicians, and long turnover times. Currently, clustered regularly interspaced short palindromic repeats (CRISPR)-based biosensors have been developed as powerful tools for nucleic acid detection. Moreover, the CRISPR method can be performed at physiological temperature, meaning that it is easy to assemble into point-of-care devices. Microfluidic chips hold promises to integrate sample processing and analysis on a chip, reducing the consumption of sample and reagent and increasing the detection throughput. This review provides an overview of recent advances in the development of CRISPR-based biosensing techniques and their perfect combination with microfluidic platforms. New opportunities and challenges for the improvement of specificity and efficiency signal amplification are outlined. Furthermore, their various applications in healthcare, animal husbandry, agriculture, and forestry are discussed.

## Introduction

Coronavirus disease 2019 (COVID-19), which was caused by severe acute respiratory syndrome coronavirus 2 (SARS-CoV-2), has resulted in more than five million global deaths to now (WHO Coronavirus Disease (COVID-19) Dashboard https://covid19.who.int (2021). In the fight against this worldwide pandemic disease, the great demands of simple, rapid, and sensitive tools for virus diagnosis promoted the rise of nucleic acid detection platforms. Quantitative PCR with reverse transcription (RT-qPCR), as a classic method for nucleic acid detection with high sensitivity and specificity, is still the gold standard (Ravi et al., 2020; Vandenberg et al., 2021). However, this method still has essential limitations due to the dependence on the accomplishment of amplification and reverse transcript processes including ∼30 cycles of denaturation, annealing, and extension steps, which could be accompanied by amplification bias and cross-contamination. Moreover, costly equipment, professional technicians, and essential turnover times are required for the thermal cycles.

Currently, clustered regularly interspaced short palindromic repeats (CRISPR) based biosensors have been developed as powerful tools for nucleic acid sensing and widely applied to the rapid diagnosis of infectious pathogens (Kellner et al., 2019; Qin et al., 2019; Broughton et al., 2020; Ding R. et al., 2021; Song F. et al., 2021; Chen et al., 2021c; Escalona-Noguero et al., 2021; Ge et al., 2021) and the detection of DNA or miRNAs associated with diseases such as cancer (Li B. et al., 2021; Wang et al., 2021c; Chen Y. et al., 2021). These biosensors rely on the CRISPR systems containing CRISPR-associated proteins (Cas) with nonspecific endonuclease activity to efficiently cleave specific targets via guide RNAs (gRNAs) (Cong et al., 2013; Mali et al., 2013; van der Oost, 2013). CRISPR systems are highly sensitive and specific; for example, Cas13 orthologs showed a ∼50-fM detection sensitivity of target RNA input (about 600,000 molecules) (Gootenberg et al., 2017). However, common CRISPR systems cannot reach clinical diagnostic demand as single-molecule sensing. The CRISPR-based biosensors are usually integrated with pre-amplification steps to boost the detection. Considering the device limitation of PCR methods, portable and isothermal amplification methods are preferred adoptions for pre-amplification and showed great potential with the capabilities to perform under easily controlled conditions, even at physiological temperature (Vincent et al., 2004; Piepenburg et al., 2006; Tomita et al., 2008). Moreover, with the advances of aptamers used in the CRISPR systems, ultrasensitive protein detection and protein/small molecular interaction identification can also be facilitated (Wang et al., 2020; Zhang K. et al., 2021; Wang et al., 2021d; White et al., 2021) (e.g., alkaline phosphatase, polynucleotide kinase/phosphatase, and chemokine).

Microfluidic platform, also known as the micro total analysis system (μTAS), has the ability to integrate sample processing and analysis on a single chip, reducing the consumption of sample and reagent and increasing the detection throughput (Manz et al., 1990; Dong et al., 2019). These features make it feasible to assemble CRISPR-based biosensors into streamlines with operational procedures and without the requirement of professional personnel, especially developed for point-of-care (POC) devices (Yin et al., 2019; Li Z. et al., 2021). This review provides an overview of recent advances in the development of CRISPR-based biosensing techniques and their perfect combination with microfluidic platforms. Furthermore, their various applications including medical diagnostics, disease screening, food-safety monitoring, and crop genotyping are outlined.

## CRISPR-Based Biosensing Techniques

CRISPR-Cas system was found to be associated with immune response in bacteria or archaea, and the genomic element, CRISPR sequence, was first discovered in *Escherichia coli* (Ishino et al., 1987). CRISPR-Cas system functioned in the adaptive immunity of microorganisms via targeting and degradation of foreign nucleic acid, so as to destroy virus invaders (Lander, 2016). There are three main stages in the CRISPR-Cas immune response: adaptation, when Cas protein recognizes and binds to the target DNA; expression, when the CRISPR array is transcribed into CRISPR RNAs (crRNAs); and interference, when crRNA guided Cas nuclease to cleave and inactivate the invading virus or plasmid genome (Makarova et al., 2020) evolutionary. In 2013, Cong et al. and Mali et al. identified that the bacterial CRISPR system can be applied in mammalian genomes engineering (Cong et al., 2013; Mali et al., 2013). They reported a type II Cas nuclease, Cas9, which can break specific double-strand DNA (dsDNA) targets via RNA-guided process and be used for directed mammalian genome editing. After that, the CRISPR system was booming in the next decade with the rapid development and improvement of efficiency, specificity, and fidelity and provided powerful tools for sophisticated genetic engineering in fundamental research and practical applications, especially in medical and agricultural fields. Moreover, components in the CRISPR system cost very low; for example, the gRNA sequence is cheap to synthesize, the quencher has a fixed sequence without the need to be redesigned and ordered for each new target, and the Cas proteins can be produced in bulk (Kellner et al., 2019). With these natural advantages, CRISPR systems were widely harnessed for biosensing approaches, blossomed in the nucleic acid detection for disease diagnoses, such as Specific High-Sensitivity Enzymatic Reporter Unlocking (SHERLOCK) (Gootenberg et al., 2017; Gootenberg et al., 2018; Kellner et al., 2019), one-HOur Low-cost Multipurpose highly Efficient System (HOLMES) (Li et al., 2018; Li L. et al., 2019), and DNA endonuclease-targeted CRISPR trans reporter (DETECTR) (Chen et al., 2018; Broughton et al., 2020).

### Available CRISPR Systems for Biosensing

Since the Cas proteins showed remarkable diversity, CRISPR systems were divided into two classes according to the effector modules composed of multiple Cas proteins (class 1, involving types I, III, and IV) or single multidomain protein (class 2, involving types II, V, and VI) (Makarova et al., 2020). Although class 1 systems extensively exist in microorganisms, their applications were limited by the need for multi-subunit effectors (Liu et al., 2021a). Thanks to the efforts on the discovery of novel systems, highly efficient and simplified CRISPR-based biosensing methods were designed. Commonly used natural Cas nucleases and their variants were described as follows.

#### Cas9

Cas9, belonging to type II systems, can induce specific double-strand breaks (DSBs) in target DNA through a dual-RNA-guided process, in which a fusion of crRNA and its base-paired trans-activating crRNA (tracrRNA) directs Cas9 to the complementary sequence (Gasiunas et al., 2012; Jinek et al., 2012). In biosensing assay, the dual-tracrRNA:crRNA can be designed as a functional artificial chimera with demand. Except for the tracrRNA:crRNA gRNAs, a proto-spacer-adjacent motif (PAM) sequence NGG (N = A, T, C, or G) was needed. When Cas9-gRNA complex recognizes PAM, the spacer of gRNA pairs with the target DNA strand to form an “R-loop” structure, after which the cleavage of DNA strands is accomplished with a blunt-end DSB 3 bp upstream of the PAM into the protospacer (Jinek et al., 2012; Cong et al., 2013; Mali et al., 2013). Cas9 has HNH and RuvC nuclease domains, which cleave the complementary strand and noncomplementary strand, respectively. When either HNH or RuvC domain was inactivated, called Cas9 nickase (nCas), only one DNA strand can be cleaved without DSBs (Anzalone et al., 2019). When both domains were inactivated, dead Cas9 (dCas9) was created, which is still able to bind to specific DNA targets but defective in nuclease activity (Qi et al., 2013). Although dCas9 cannot cleave target DNA like wild-type Cas9, it provides a scaffold for recruiting effectors to specific sites.

A major barrier to the applications of CRISPR is the “off-target” effects, referring to the cleavage at unintended sites because of the nuclease tolerating mismatches between gRNA and off-target DNA (Liu et al., 2021a). To deal with this issue, marked improvements were made in gRNA modification, protein and guide engineering, and novel enzymes screening (Tycko et al., 2016). For example, truncated gRNAs (Tru-gRNAs) with shorter regions of target complementarity <20 nucleotides in length (Fu et al., 2014), hp-sgRNAs designed with a hairpin onto the spacer region (Kocak et al., 2019), and CRISPR hybrid RNA-DNA (chRDNA) guides (Donohoue et al., 2021) have been reported to effectively optimize Cas9 specificity and preserve on-target activity. Nuclease engineering, such as using paired nCas9 nickases or fusing dCas9 with the FokI nuclease domain, can also improve specificity (Guilinger et al., 2014; Shen et al., 2014; Tsai et al., 2014). Another strategy is to screen novel high-fidelity Cas9 variants that are developed by rational design or directed evolution (Kim et al., 2020; Schmid-Burgk et al., 2020), providing useful instructions to their application in different situations.

Despite the robust activity, the specific requirement of PAM in DNA targeting limits the flexibility and applications of Cas9 (Anzalone et al., 2019). Ongoing efforts were made to realize PAM-free nucleases through natural ortholog mining and protein engineering (Collias and Beisel, 2021).

#### Cas12

Cas12, a kind of type V nuclease, contains a RuvC-like domain only and cleaves both target and non-target strands, introducing a staggered DNA DSB (Zetsche et al., 2015). Cas12a (first reported as Cpf1) processes pre-crRNA into mature crRNA and cleave target DNA independent of additional RNA species, greatly simplifying the editing design (Zetsche et al., 2015; Zetsche et al., 2017). Cas12a has short T-rich PAM recognition sites, rather than the G-rich PAM following the target DNA for Cas9, expanding the targeting range (Zetsche et al., 2015). Cas12b (formerly known as C2c1) contains RuvC-like endonuclease domains distantly related to Cas12a but depends on both crRNA and tracrRNA for DNA cleavage (Strecker et al., 2019; Ming et al., 2020). Although Cas12a offers unprecedented flexibility, more compact versions are explored and engineered to extend the application. For example, Cas12c, Cas12h, and Cas12i have been identified with RNA-guided dsDNA interference activity (Yan et al., 2019). Cas12g was characterized by RNA-guided collateral ribonuclease and single-strand deoxyribonuclease activities (Yan et al., 2019). Cas12f (also known as Cas14) nucleases cleave single- and dsDNA targets triggered by a 5′ T- or C-rich PAM sequence (Harrington et al., 2018; Karvelis et al., 2020; Bigelyte et al., 2021; Xu X. et al., 2021; Takeda et al., 2021).

#### Cas13

Cas13 belongs to type VI CRISPR-Cas systems and contains two Higher Eukaryotes and Prokaryotes Nucleotide-binding (HEPN) domains (Shmakov et al., 2015). Cas13a, which was initially named C2c2, exhibits a collateral cleavage effect and only requires single-guide crRNA (Abudayyeh et al., 2016). After target binding, the HEPN catalytic site of Cas13a protein is activated, which subsequently cleaves both single-stranded target and collateral RNAs in a non-specific manner (Liu et al., 2017). Due to the “collateral effect,” Cas13a can be promisingly used for nucleic acid detection, not only RNA but also dsDNA (Abudayyeh et al., 2017; Gootenberg et al., 2017; Wang et al., 2019). Currently, various Cas13 orthologs and variants were identified and characterized, including Cas13b (Smargon et al., 2017), Cas13d (Yan et al., 2018), Cas13X and Cas13Y (Xu C. et al., 2021), and Cas13bt (Kannan et al., 2021). In addition, the development of unrelated CRISPR nucleases, such as Csm6 combined with Cas13, showed a robust detection of RNA targets (Liu T. Y. et al., 2021).

**FIGURE 1:**
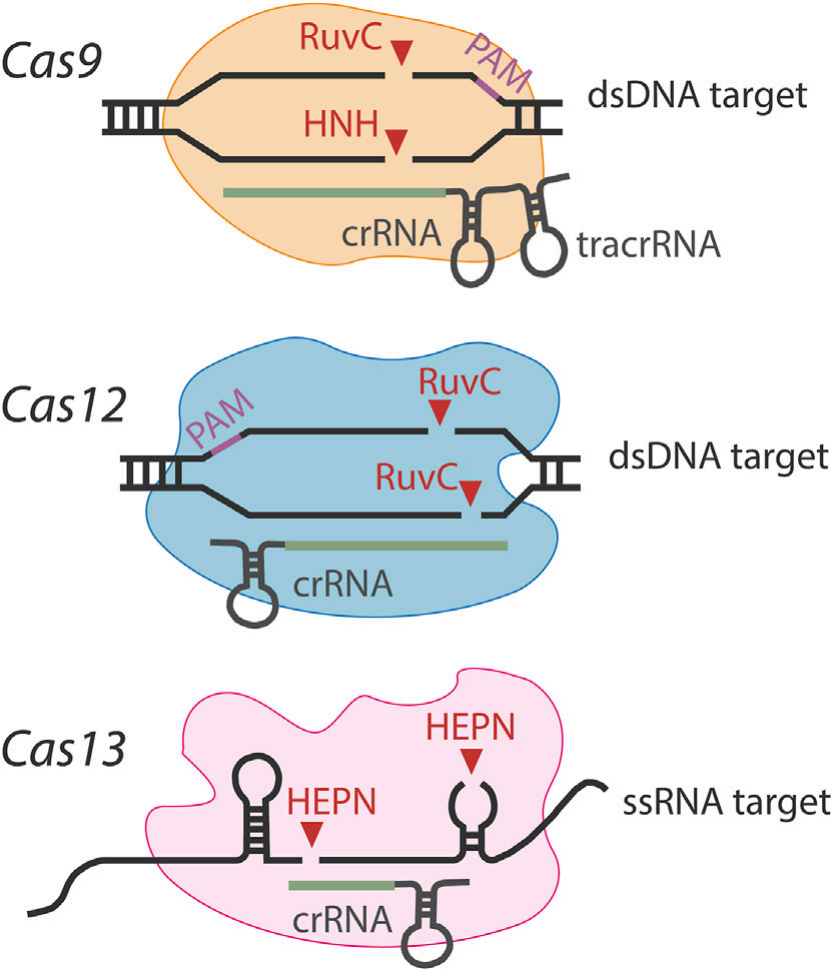
Diagrams of Cas9, Cas12, and Cas13 nucleases.

Already, these Cas nucleases have been used for biosensing purposes, and different effectors show different nucleotide cleavage preferences (as shown in Figure 1), which can be selected according to specific applications. Major features of these most used Class II Cas nucleases are listed in Table1. Moreover, continued exploration is ongoing to uncover new functional orthologs and thoroughly investigate the targeting rules of Cas nucleases, leading to a substantial increase in the efficiency of target cleavage and high-target specificity.

**TABLE 1:** Main features of most used Class II Cas nucleases.

Nucleases	Type	Nuclease domain	Guide type	Target	*trans*-Cleavage	PAM sequence
Cas9	Type II	RuvC and HNH	crRNA and tracrRNA	dsDNA	-	G-rich
Cas12	Type V	RuvC-like	crRNA only/crRNA and tracrRNA	Mostly dsDNA	dsDNA/ssDNA	T-rich^a^
Cas13	Type VI	HEPN	crRNA only	RNA	ssRNA	-

a T- or C-rich for Cas12f, also known as Cas14.

### Consideration for Pre-Amplification Strategies

Generally, pre-amplification steps were needed for samples with very low target concentration, which are difficult to distinguish from the background. Traditional PCR methods are highly sensitive and specific but demand costly equipment and professional technicians, as well as the cost of essential turnover times for the thermal cycles. Thus, portable and isothermal amplification methods have been developed, including helicase-dependent amplification (HDA) (Vincent et al., 2004), recombinase polymerase amplification (RPA) (Piepenburg et al., 2006), and loop-mediated isothermal amplification (LAMP) (Tomita et al., 2008). HDA uses a DNA helicase to separate dsDNA and generate single-stranded templates for primer hybridization by single-stranded DNA (ssDNA)-binding proteins (SSBs), which was subsequently extended by DNA polymerases. In the whole process, the initial heat denaturation and subsequent thermocycling steps required by PCR can all be omitted, which provides a simple DNA amplification using low temperature from the beginning to the end of the reaction (Vincent et al., 2004). Despite its simplicity, the specificity of HAD cannot be comparable to PCR, which hampered the utilities. RPA mainly relies on three core enzymes: a recombinase, which is capable to pair oligonucleotide primers; an SSB, which binds to displaced strands of DNA to prevent the primers from being displaced; and strand-displacing polymerase (Piepenburg et al., 2006; Ortiz et al., 2022). Once the recombinase-driven primer targeting of homologous sequence in dsDNA is completed, DNA synthesis begins with the strand displacing polymerase, achieving exponential amplification. Similar to HAD, RPA steps can all be performed at a constant optimal temperature of 37°C, which can also hold the activities at room temperature. A key obstacle for the use of RPA is the design for amplification primers and probes, which are usually longer than general PCR primers and need to be optimized by customers on their own conditions. LAMP uses a polymerase with high strand displacement activity and a replication activity and adopts two or three sets of primers to increase specificity and an additional pair of “loop primers” to accelerate the reaction (Tomita et al., 2008). In LAMP, the target sequence is amplified at a constant temperature of 60°C–65°C, which can be easily obtained through a water bath.

Recently, novel isothermal amplification methods have been developed and proposed for sensitive detection. Rolling circle amplification (RCA) is initiated with circular template ligation, forming a long ssDNA or RNA, followed by primer-induced ssDNA elongation (Nosek et al., 2005; Ali et al., 2014). At constant temperature (room temperature to 65°C), a large number of repeats complementary to the circular template can be produced. Additionally, RCA can be conducted in both free solutions and on top of immobilized targets (solid-phase amplification). With these advantages, CRISPR/Cas12a- or CRISPR/Cas13a-triggered RCA is developed as a highly sensitive and specific biosensor, for example, for the detection of microRNAs (Li D. et al., 2020; Tian et al., 2020; Qing et al., 2021; Zhou et al., 2021). Alternative isothermal amplification approaches, such as exponential amplification reaction (EXPAR) (Song J. et al., 2021; Wang et al., 2021b), hybridization chain reaction (HCR) (Xing et al., 2020; Kachwala et al., 2021; Liu et al., 2022), and strand displacement amplification (SDA) (Chen et al., 2021b; Deng et al., 2021; Gong et al., 2021) were combined with CRISPR systems for sensing pathogenic bacteria and viruses, gene mutation, and even proteins. Different characteristics of this isothermal amplification method were compared as listed in Table 2.

**TABLE 2:** Comparison of isothermal amplification methods.

Methods	Temperature (°C)	Time	Involved proteins	Primers
HDA	∼37	~2 h	DNA helicase, SSB, DNA polymerase	2
RPA	25–42 (optimal 37)	20 min∼1 h	Recombinase, SSB, strand-displacing polymerase	2
LAMP	60–65	20 min∼2 h	Strand-displacing DNA polymerase	6–8
RCA	Room temperature to 65	∼2 h	DNA polymerase	4
EXPAR	∼37	2∼3 h	DNA polymerase	2
HCR	∼37	∼1 h	-	-
SDA	37–60	0.5∼1 h	Strand-displacing DNA polymerase, nicking endonuclease	4

Note. HDA, helicase-dependent amplification; RPA, recombinase polymerase amplification; LAMP, loop-mediated isothermal amplification; RCA, rolling circle amplification; EXPAR, exponential amplification reaction; HCR, hybridization chain reaction; SDA, strand displacement amplification; SSB, single-stranded DNA-binding protein.

Additionally, different kinds of improvements have been tried to develop amplification-free CRISPR-based biosensors with the assistance of Au nanoparticles (Choi et al., 2021), gold nanoclusters (Liu P.-F. et al., 2021), nanopores (Nouri et al., 2020), and graphene field-effect transistor (Hajian et al., 2019). Another sensitive strategy without target amplification was reported by making use of both Cas13a and Cas14a (Sha et al., 2021). Alternatively, microfluidics techniques provided tremendous potentials for amplification-free platforms, including droplet manipulation and on-chip integration (Bruch et al., 2019a; Bruch et al., 2021; Yue et al., 2021b; Tian et al., 2021), which will be highlighted in the next section.

## Powerful Support by Microfluidics Platforms

An ideal biosensor for molecular detection should be accurate, sensitive, and capable to give results rapidly (Goode et al., 2015). In this regard, minimal sample extraction and optimized preparation procedures will help to improve the detection sensitivity without increasing the cost of overall assay time. Microfluidics means precisely controlling and manipulating the behavior of fluids on a geometrically constrained small scale (Dong et al., 2019). The introduction of microfluidic technology can get rid of the demand for bulky instrumentation and offers ingenious storage for the required reagents, which attracts more and more interest for the development of biosensors with promising potential to transform into POC devices.

### Optimized Sample Preparation and Readout

Most biosensors typically require a “two-step” assay with target amplification reactions and detection steps separately, which brings some drawbacks with the risk of carryover contamination (Yue et al., 2021a). To solve this problem, sophisticated assemblies were introduced, for example, pre-embedding CRISPR-Cas12a reagents on the inner wall of the tube cap and then mixing with amplicon solution by hand shaking (Chen et al., 2020c). Another group carried out RPA reaction and CRISPR-Cas12a detection in spatially separated but connected phases in one pot (Yin et al., 2020). Similarly, Wu et al. used an oil-sealed polypropylene (PP) bag with three chambers for washing and amplification/detection (Wu et al., 2021a).

Microfluidic chips hold promises to streamline analysis steps and integrate them in closed microchannels separately, with the capability of reducing reagent consumption and increasing detection throughput (Ahmadi et al., 2020). For example, droplet microfluidics enables to confine the Cas catalysis in cell-like-sized reactors via an ultralocalized droplet, enhancing the local concentrations of target and reporter simultaneously to obtain excellent specificity and sensitivity (Yue et al., 2021b; Tian et al., 2021). Lab-on-a-chip can also be introduced, which implements a miniaturized system, spatially separating multiple immobilization areas within a single channel (Bruch et al., 2019a; Bruch et al., 2021). Taken together, sealing up spatially separately helps to increase the stability for long-term storage, and the automated process makes it more convenient to extend the applications with a reduced cost of operation time.

### Development for Automated POC Devices

According to the WHO guidelines, POC testing needs to be affordable, sensitive, specific, user-friendly, robust and rapid, equipment-free, and deliverable to all people who need the test (ASSURED) (van Dongen et al., 2020). Taking these into account, microfluidics techniques promisingly offer simplified approaches (sample-in result-out) for biosensing automatedly with high throughput. Chen et al. integrated the CRISPR/Cas12a system and recombinase-aided amplification in a centrifugal microfluidic device, avoiding the catalysis of Cas12a to the template DNA. This Cas12a-assisted straightforward microfluidic equipment for analysis of nucleic acid, termed CASMEAN, was reported to enable nucleic acid detection within 1.5 h (Chen et al., 2020b). Wu et al. developed a reversible valve-assisted chip to integrate CRISPR/Cas12a system and LAMP into a single chip, which has three reversible rotary valves and can be rotated relying on the direction-dependent Coriolis pseudo force. Their POC device achieves a limit of detection (LoD) of 30 copies/reaction for the detection of *Vibrio parahaemolyticus* (Wu et al., 2021b). Chen et al. proposed a POC biochip with preloaded CRISPR/Cas12a reagents for processing automatedly, capable to detect the genotypes within 20 min (Chen Y. et al., 2021). Taken together, microfluidics strategies facilitate biosensors developing to POC device development.

## Applications of CRISPR-Based Biosensing Techniques

Nowadays, CRISPR systems have been established as powerful biosensing tools for detecting various targets (Li et al., 2019b; Bao et al., 2021; Kaminski et al., 2021). Several developed CRISPR-based biosensors were characterized in Table 3. Their main application in the sensing of a wide range of molecular targets was highlighted as follows.

**TABLE 3:** Major characteristic of several developed CRISPR-based biosensors for nucleic acid detection.

Name	Cas systems	Target	Amplification	Readout	Sensitivity	Specificity	Time	Ref
SHERLOCK	Cas13a	DNA/RNA	RPA	Fluorescent	aM	1 nt	<2 h	Gootenberg et al., 2017; Kellner et al., 2019
SHERLOCKv2	PsmCas13b, LwaCas13a, CcaCas13b, AsCas12a	DNA/RNA	RPA	Fluorescent/colorimetric	zM	1 nt	0.5–3 h	Gootenberg et al. (2018)
SHERLOCK + HUDSON	Cas13a	DNA/RNA	RPA	Fluorescent	aM	1 nt	<2 h	Myhrvold et al. (2018)
DETECTR	Cas12a	DNA	RPA	Fluorescent	aM	6 nt	∼2 h	Broughton et al. (2020); Chen et al. (2018)
HOLMES	Cas12a	DNA/RNA	PCR	Fluorescent	aM	1 nt	∼1 h	Li et al. (2018)
HOLMESv2	Cas12b	DNA/RNA	LAMP/PCR	Fluorescent	aM	1 nt	∼1 h	Li et al. (2019a)
SHINE	Cas13	RNA	RPA	Colorimetric	aM	-	50 min	Arizti-Sanz et al. (2020)

Note. RPA, recombinase polymerase amplification; LAMP, loop-mediated isothermal amplification.

### Diagnosis of Pathogen Infections

#### SARS-CoV-2

The main focus of CRISPR-based biosensing techniques has been the diagnostics of pathogen infection benefits from their DNA- and RNA-targeting nucleases (Escalona-Noguero et al., 2021). Amid the ongoing pandemic of COVID-19, enormous efforts have been paid for the detection of SARS-CoV-2 (Nouri et al., 2021). SHERLOCK was first developed using Cas13a with RPA amplification to detect Zika and Dengue virus and to identify human DNA, exhibiting similar levels of sensitivity to RT-qPCR (capable of single-molecular detection) (Gootenberg et al., 2017). Subsequently, it was advanced to SHERLOCK version 2 (SHERLOCKv2) integrated with multiplexed orthogonal CRISPR enzymes to increase signal sensitivity and portable lateral-flow readout (Gootenberg et al., 2018). SHERLOCK was further advanced with HUDSON (heating unextracted diagnostic samples to obliterate nucleases) for viral detection directly from bodily fluids, enabling instrument-free virus detection directly from patient samples within less than 2 h (Myhrvold et al., 2018). The step-by-step instructions for SHERLOCK assays were then published, demonstrating the use of Cas13 or Cas12, combination with isothermal pre-amplification, and the detection through fluorescence and colorimetric readouts (Kellner et al., 2019). As the outbreak of SARS-CoV-2, it was streamlined into SHERLOCK testing in one pot (STOP) combined with simplified extraction of viral RNA, which can be performed for the detection of SARS-CoV-2 at a single temperature in less than 1 h and with minimal equipment (Joung et al., 2020a; Joung et al., 2020b). SHERLOCK-based assay for SARS-CoV-2 detection has been tested in a validated clinical cohort including 154 nasopharyngeal and throat swab samples and 380 SARS-CoV-2-negative preoperative samples (Patchsung et al., 2020). Results showed that it reached a detection limit of 42 RNA copies per reaction, with 100% sensitivity for fluorescence readout, 97% sensitivity for lateral-flow readout, and both 100% specificity. DETECTR provided an attomolar sensitivity for DNA detection using Cas12a and was optimized to detect SARS-CoV-2 via its N (nucleoprotein) and E (envelop small membrane protein) genes (Chen et al., 2018; Broughton et al., 2020). DETECTR can be performed in less than 40 min from respiratory swab RNA extracts using lateral flow readout and be validated by 36 patients with COVID-19 infection and 42 patients with other viral respiratory infections with 95% positive predictive agreement and 100% negative predictive agreement with RT-qPCR assay from the US Centers for Disease Control and Prevention (Broughton et al., 2020). Another Cas13a-based assay, Streamlined Highlighting of Infections to Navigate Epidemics (SHINE), was developed to detect SARS-CoV-2 RNA from unextracted samples by optimizing RPA-based pre-amplification and Cas13-based detection into a single step and improve HUDSON to accelerate viral extraction in nasopharyngeal swabs and saliva samples (Arizti-Sanz et al., 2020). The validation from 50 nasopharyngeal patient samples demonstrated that SHINE has 90% sensitivity and 100% specificity against RT-qPCR with a sample-to-answer time of 50 min.

To balance the sensitivity, specificity, and test availability, a variety of strategies have been tried to improve the detection of SARS-CoV-2. For example, DNA-modified gold nanoparticles (AuNPs) were utilized for a universal colorimetric readout according to the change in the surface plasmon resonance, which can be monitored by UV-vis absorbance spectroscopy facially and observed by the naked eye (Zhang W. S. et al., 2021). An alternative is assisted with magnetic AuNP probe colorimetric assay, which is not dependent on sophisticated instruments and can be potentially adopted under poor conditions (Jiang et al., 2021). Different amplification strategies were also explored to couple with CRISPR-based SARS-CoV-2 biosensing, such as HCR amplification, which was reported in an evanescent wave fluorescence biosensing platform providing an attomolar detection level towards SARS-CoV-2 within 1 h (Yang et al., 2021), and multiple cross displacement amplification (MCDA), which conducts reverse transcription MCDA reaction when CRISPR-Cas12a/CrRNA complex recognizes the predefined target sequences and subsequently degrades a single-strand DNA to confirm the target detection (Zhu et al., 2021). A major direction of biosensing platforms is to develop POC devices. Hence, portable approaches were taken into mind. An attempt was made to adopt the available personal glucose meter to readout for quantitative detection of SARS-CoV-2 via converting the virus signal to a glucose-producing reaction (Huang et al., 2021). Already benefitting from an elegant detection mechanism, fast assay time, and low reaction temperature, these assays can be further advanced via integration with powerful, digital-based detection. A coined digitization-enhanced CRISPR/Cas-assisted one-pot virus detection (deCOViD) was reported to achieve qualitative detection in <15 min and quantitative detection in 30 min with down to 1 genome equivalent (GE) per µl of SARS-CoV-2 RNA and 20 GE per µl of heat-inactivated SARS-CoV-2 (Park et al., 2021). A digital warm-start CRISPR (dWS-CRISPR) assay showed a detection down to five copies/μl SARS-CoV-2 RNA, as well as the capability to directly detect SARS-CoV-2 in heat-treated saliva samples without RNA extraction (Ding X. et al., 2021). These digital methods facilitate accurate, sensitive, and reliable CRISPR assays into POC devices, with a high signal-to-background ratio and broad dynamic range. In addition, preventing amplicon-formed aerosol contamination is also an important process. One group pre-added reagent on the inner wall of the tube lid, which were then hand-shaken to make them flow into the tube and mix with amplicon solution, which could be processed within 40 min and reach a sensitivity of 20 copies RNA of SARS-CoV-2 (Chen et al., 2020c). A more portable platform incorporated sample preparation with the facile magnetic-based operation of nucleic acid concentration and transport and streamlined into a compact palm-sized thermoplastic cartridge functioning in a fully integrated and autonomous way, which can detect 1 GE/μl SARS-CoV-2 RNA from 100 μl of sample in less than 30 min (Chen F.-E. et al., 2021). This kind of integration of microfluidic platforms streamlined sample preparation procedures fully into autonomous and portable devices, opening a new avenue to facilitate POC methods for SARS-CoV-2 detection (Mu et al., 2020; Ramachandran et al., 2020; Basiri et al., 2021).

#### Other Infectious Bacteria and Viruses

CRISPR-based methods have been widely developed for the detection of infectious pathogens. Detection of HIV-1 termed as Solid-State CRISPR-Cas12a-Assisted Nanopores (SCAN) can recognize target DNA concentrations at least to 10 nM within 1 h, without the requirement of pre-amplification steps (Nouri et al., 2020). A report detecting HBV based on DETECTR showed an LoD of 1 copy/μl within 13 min using fluorescent readout; however, the LoD of lateral flow test strip technique costing 20 min was not shown (Ding R. et al., 2021). Utilizing lateral flow, a Cas12a-based biosensor was designed for the detection of Epstein–Barr virus (EBV), achieving a sensitivity of 7.1 × 10^–14^ mol/L (approximately 42,000 copies per μl) (Yuan et al., 2020). Another Cas12a-based biosensor using a dynamic aqueous multiphase reaction system was introduced to detect human papillomavirus (HPV) with sensitivities of 10–100 copies in less than 1 h (Yin et al., 2020). Supported by automated microfluidic mixing, an approach for Ebola virus detection was established using Cas13a and achieved an LoD of 20 pfu/ml (5.45 × 10^7^ copies/ml) of purified Ebola RNA within 5 min (Qin et al., 2019).

Cas12a-based biosensing was also developed for the detection of a variety of pathogenic microorganisms, such as *Listeria monocytogenes* (Li F. et al., 2021), *Cryptosporidium parvum* (Yu et al., 2021), *Salmonella* (Ma et al., 2021), *Helicobacter pylori* (Qiu et al., 2021), *Yersinia pestis* (You et al., 2021), *E. coli*, and *Staphylococcus aureus* (Bonini et al., 2021). Coupling with a reversible valve-assisted chip, sample preparation, Cas12a reactions, and LAMP was integrated and controlled precisely to perform the detection of *V. parahaemolyticus*, achieving an LoD of 30 copies/reaction within 50 min (Wu et al., 2021b). Cas14a (also known as Cas12f1) was exploited for the detection of pathogenic bacteria combined with a universal nucleic acid magneto-DNA nanoparticle system, which can achieve 1 cfu/ml or 1 aM sensitivity (Song F. et al., 2021). The Cas14a1-mediated platform was also reported for the detections of pathogens benefitting from its small size and independence of PAM (Ge et al., 2021). Further development by introducing novel aptamer, a Cas13a assay enables mix-and-read detection of viable pathogenic bacteria without the need of reverse transcription, nucleic acid amplification, and chemical labeling, which obtained an LoD of 10 CFU for *Bacillus cereus* (Zhang T. et al., 2021).

### Detection of Non-Infectious Diseases

CRISPR-based biosensors also showed promising potential for the detection of non-infectious human diseases, such as cancer, based on the features with disease-related gene mutations, single-nucleotide polymorphism, DNA methylation, and so on (Aman et al., 2020; Li B. et al., 2021; Wang et al., 2021c; Chen Y. et al., 2021; Sheng et al., 2021). For example, a Cas12a-based biosensor showed sensitive detection of gene-PIK3CA^H1047R^ mutation low at 0.001%, which has great potential to predict early-stage breast cancer (Deng et al., 2021). A Cas12a-based transcription factor detection method showed an LoD of 0.2 pM for NF-kappaB p50 subunit from cancer cell samples, which can be further applied for physical dysfunction monitoring and drug screening (Li B. et al., 2021). Recent work introduced a hairpin probe to preserve the analytical fidelity and developed a CRISPR/Cas9-triggered hairpin probe-mediated biosensing method, termed the CHP system, specifically initiating double isothermal amplifications when Cas9-mediated cleavage occurs and the hairpin probe recognizes the original sequences (Wang M. et al., 2021). This system has an LoD at the attomole level to quantify DNA targets and identify single-nucleotide variations with allelic fractions down to 0.01%–0.1%.

In addition to the genome profile, other disease-related nucleic acids were chosen as detection targets. MicroRNAs (miRNAs), which have been reported to be related to many biological processes, are regarded as disease biomarkers. Hence, Cas12a or Cas13a systems have been widely used for the profiling of microRNAs, with or without assisting pre-amplification, and can highly reach an fM sensitivity and single-base specificity (Tian et al., 2020; Cui et al., 2021; Sha et al., 2021; Zhou et al., 2021). Circulating tumor DNA (ctDNA) represented another type of credible biomarkers for clinical diagnosis and prognosis. In this regard, a CRISPR/Cas9 biosensor based on a 3D graphene/AuPtPd nanoflower was developed to trigger entropy-driven strand displacement reaction for ctDNA detection (Chen et al., 2020a).

### Testing in Animal Husbandry, Agriculture, and Forestry

In socioeconomic terms, CRISPR-based biosensing techniques were also developed to satisfy the demand from animal husbandry, agriculture, and forestry. For example, a Cas12a-based lateral flow biosensor combined with PCR amplification was used for the detection of the African swine fever virus (ASFV), achieving a sensitivity of 2.5 × 10^–15^ M within 2 h from swine blood (Wu et al., 2020). Another Cas12a-based reversible valve-assisted chip was established for the rapid detection of *V. parahaemolyticus* for the seafood test, with an LoD of 30 copies/reaction by using 600 μl of samples (Wu et al., 2021b). In the field of agriculture and forestry, the Cas9 system combined with AuNPs has been developed to identify plant-associated disease through the detection of *Phytophthora infestans* (Chang et al., 2019). Cas12a-based biosensors were developed for the detection of plant DNA virus (Mahas et al., 2021) and the nopaline synthase terminator in genetically modified crops (Huang et al., 2020).

### Sensing for Non-Nucleic Acid Targets

As shown above, robust development of CRISPR-based biosensors was applied to detect the nucleic acid targets. Combined with a microfluidic chip, the CRISPR/Cas9 system can be developed for rapid and efficient kinase screening and further to separate cells according to different deformability, with flexible cells flowing out and stiff cells remaining trapped (Han et al., 2016). Recently, immunoassays based on CRISPR/Cas systems for the sensitive and rapid detection of protein targets were also explored, which is akin to the traditional ELISA (Zhao Q. et al., 2021; Li H. et al., 2021). Li et al. designed a series of aptamer-flanked activator DNA strands to correlate non-nucleic acid analytes with the CRISPR/Cas12a system, enabling an ultrasensitive detection (Li H. et al., 2021). Zhao et al. employed DNA-AuNPs to establish the signal transduction between trans-cleavage of CRISPR/Cas12a and protein analytes and showed a quantitative level of attomolar, 1,000-fold more sensitive and 15-fold wider detection range than traditional ELISA (Zhao Q. et al., 2021). CRISPR-based electrochemiluminescence biosensors have been explored for the detection of enzymes like polynucleotide kinase/phosphatase (Wang et al., 2020) and alkaline phosphatase (Wang et al., 2021d), and signal factors like endogenous chemokine (White et al., 2021) and sialic acid-binding immunoglobulin-like lectins (Zhang K. et al., 2021). Similar to aptamers, DNAzymes or DNA ligations were also designed to introduce CRISPR/Cas12a biosensor for non-nucleic acid targets, for example, testing melamine (Qiao et al., 2021), metal ion Na^+^ (Li C.-Y. et al., 2020), NAD^+^, and ATP (Zhao J. et al., 2021; Niu et al., 2021). Additionally, with the adoption of AuNP, the applications of CRISPR-driven biosensors were potentially extended, such as the ultrasensitive detection of mycotoxins including aflatoxin M1 (Abnous et al., 2021), assessment for telomerase activity by analyzing telomeric repeat DNA, and internal control products (Cheng et al., 2021). Furthermore, CRISPR/Cas12a-based biosensors can monitor protein/small molecule interactions, like streptavidin/biotin and anti-digoxigenin/digoxigenin (Kim et al., 2021).

## Conclusion and Perspectives

**FIGURE 2:**
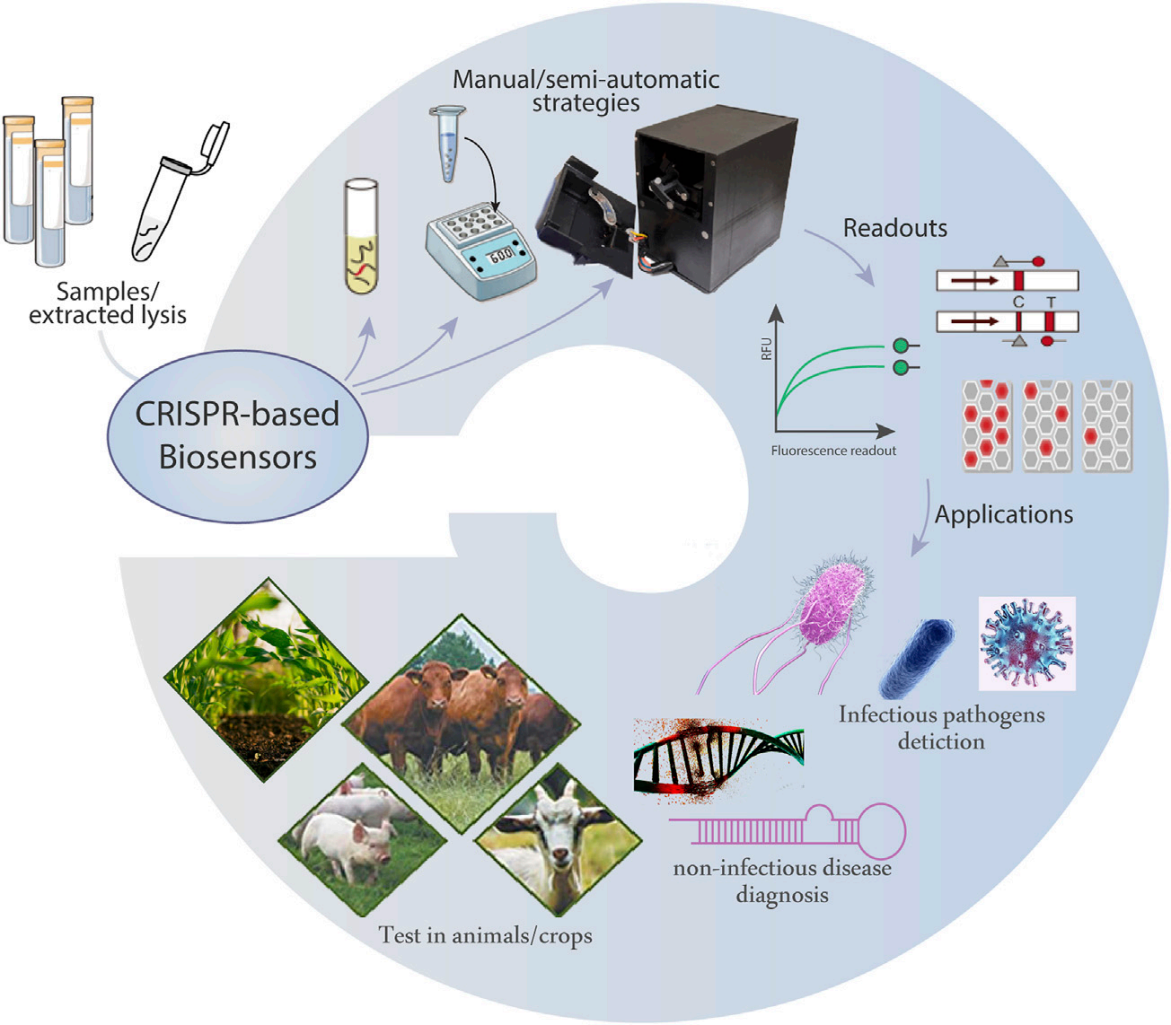
Illustration of the wide applications of CRISPR-based biosensors.

The CRISPR-based biosensors provide low-cost and easily scalable tools for the detection of various targets with high sensitivity and specificity. The combinations with microfluidic techniques powerfully integrate multiple steps of sample preparation, Cas-mediated catalysis, target amplification, and readout. Currently, these biosensors have been applied in nucleic acid-based diagnostics, protein tests, metal ion monitoring, and protein/small molecule interactions screening, which are promising in the fields of healthcare, animal husbandry, agriculture, and forestry (Figure 2).

Additionally, these platforms still face challenges in the multiplex detection, due to the limited signal reporting strategies and possible cross-reactions, which would be introduced with the interference between recognition molecules and various analytes, and exacerbated by the complexity of clinical samples (Bruch et al., 2019b; Li et al., 2019c). Introducing various labels, including fluorophore dyes, enzymes, or beads, and effectively combining different strategies will be promising directions. SHERLOCKv2 made four-channel single-reaction multiplexing of Cas12a and Cas13, increasing the signal sensitivity by introducing an auxiliary CRISPR-associated enzyme Csm6 (Gootenberg et al., 2018). Another successful attempt used an orthogonal DNA/RNA collateral cleavage by Cas12a and Cas13a assay in a single tube simultaneously, which specifically illuminated two spectral differentiated DNA and RNA probes, respectively, exhibiting 100% sensitivity and specificity for clinical samples analysis (32 swab specimens for SARS-CoV-2 and 35 ASFV-suspected swine blood samples) (Tian et al., 2022). Furthermore, thinking of machine learning approaches and the internet for wireless signal transmission over the cloud supports futuristic decision making (Ibrahim et al., 2020).
